# Sweet taste loss in myasthenia gravis: more than a coincidence?

**DOI:** 10.1186/1750-1172-9-50

**Published:** 2014-04-11

**Authors:** Joelle N Chabwine, Muriel V Tschirren, Anastasia Zekeridou, Basile N Landis, Thierry Kuntzer

**Affiliations:** 1 Department of Clinical Neurosciences, Lausanne University Hospital (CHUV), Rue du Bugnon 46, 1011 Lausanne, Switzerland; 2 Department of Otorhinolaryngology-Head and Neck Surgery, Geneva University Hospital, Geneva, Switzerland

**Keywords:** Dysgeusia, Sweet taste, Myasthenia gravis, Thymoma

## Abstract

Sweet dysgeusia, a rare taste disorder, may be encountered in severe anti-acetylcholine receptor antibody (AChRAb)-myasthenia gravis (MG). A 42 year-old man reported progressive loss of sweet taste evolving for almost 10 weeks, revealing an AChRAb-positive MG with thymoma. Improvement of sweet perception paralleled reduction of the MG composite score during the 15 months follow up period, with immunosuppressive and surgical treatments. We suggest that sweet dysgeusia is a non-motor manifestation of MG that may result from a thymoma-dependent autoimmune mechanism targeting gustducin-positive G-protein-coupled taste receptor cells, in line with recent data from MRL/MpJ-Fas^lpr^/ (MRL/lpr) transgenic mice with autoimmune disease.

## Letter to the editor

Myasthenia Gravis (MG) is an autoimmune disorder characterized by fluctuating muscle weakness and ocular or bulbar signs [1]. Non-motor symptoms may however develop, including taste disorders, which concern 5% of cases. In these patients, thymoma is common and associated with severe MG [2,3]. Here, we describe the kinetics of sweet taste loss related to a mild anti-acetylcholine receptor antibody (AChRAb)-positive MG and its recovery along with disappearance of myasthenic symptoms under treatment. In light of the literature, this case further underlines the particular association of selective sweet taste disorder with thymoma-associated MG, our hypothesis being the coexistence of an autoantibody selectively targeting G-protein-coupled receptor cells (GPCRs).

## Case report

**Figure 1:**
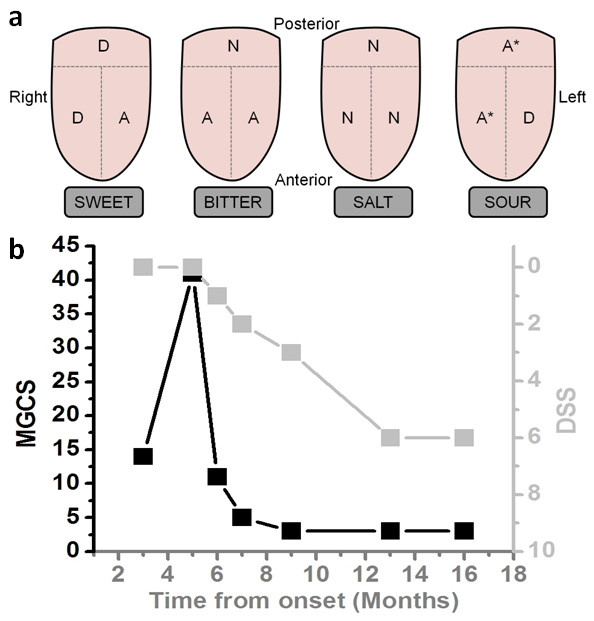
**Taste evaluation and variation of the dysgeusia according to treatment. a** (top panel). Taste evaluation performed 3 months after loss of sweet taste, conducted as shown, on the left and right of the anterior part of the tongue and globally on the posterior part. Perception of sweet was overall impaired and salt perception was normal. Bitter was only perceived at the posterior part, while sour test results changed from one evaluation to another (*). N: normal perception; D: diminished; A: abolished. **b** (bottom panel). Variations of the dysgeusia subjective score (DSS, light gray squares, right ordinates) according to MG composite score (MGCS, black squares, left ordinates) during the 16 months follow-up period. MG score improved in parallel with recovery in sweet perception for one year, then both scores stabilize, with an almost normal MGCS and a 60% of normal sweet perception. The high MGCS at month 5 corresponds to the MG crisis after thymoma removal.

A 42 year-old healthy man complained of progressive and prominent loss of sweet taste with mild salt taste impairment evolving over ten weeks. On examination, olfactory and trigeminal functions were normal. Taste function was absent for sweet, impaired for bitter, uncertain for sour and normal for salt (Figure 1a). Brain MRI and CSF analysis were normal. The usual causes of taste disturbance were excluded [4], including normal serum zinc levels. Six weeks after taste disorders onset, the patient reported fluctuating diplopia worsening in the evenings, correlated with variable gaze abnormalities on consecutive evaluations, no muscle weakness, but general fatigue. Therefore, investigations were undertaken, displaying a 20% surface decrement upon 3-Hz repetitive nerve stimulation of the accessory nerve to upper trapezius muscle, and elevated serum AChRAb level (114 pmol/ml, normal < 0.2), confirming a MGFA class I MG [5]. Pyridostigmine improved most of the symptoms including dysgeusia. A large infiltrating thymoma (80% B2 and 20% B3 in WHO classification, pT2 N0 Mx and modified-Masaoka-Conga type IIa) was discovered in the chest CT-scan, and surgically removed with consecutive myasthenic crisis requiring 2 weeks ICU care. Under immunosuppressive therapy (azathioprine shifted to mycophenolate mofetil due to liver toxicity) associated to prednisone and pyridostigmine, the patient gradually recovered. Fifteen months clinical follow up showed MG composite score improvement from 14 to 3/41 and a sweet dysgeusia numeric scale progress (dysgeusia subjective scale, DSS:0; lack of perception to 10; normal perception) from 0 to 6/10 (Figure 1b). Psychophysical olfactory and gustatory testings [6] were then normal. The patient enjoyed sweet foods again and could return to work.

## Discussion

Taste impairment (mainly sweet dysgeusia) is rare in MG [2,3] and remains a very unusual presentation of taste disorders [7]. Within the taste buds, GPCRs are responsible for sweet, bitter and umami taste detection whereas salty and sour transduction is GPCR-independent [8]. The selective sweet taste deficit in MG suggests therefore dysfunction in GPCR-containing receptor cells. Although most of GPCRs are narrowly tuned to a specific taste modality, some correspond to more than one taste [9], possibly explaining additional taste perception deficits in MG.

Sweet dysgeusia always coexists with thymoma in MG, suggesting a paraneoplastic phenomenon corroborated by its repeated description as first sign of lung cancer [10]. However, sweet taste disappears in MG whereas it is distorted in lung cancer [10]. In our patient, dysgeusia coexisted with mild myasthenic symptoms and recovered in parallel with them under treatment [2]. Moreover, in all reported MG-associated dysgeusia cases (usually severe), both AChRAb and thymoma were present, and dysgeusia evolved in parallel with AChRAb titers [11]. We conclude that dysgeusia shared a common pathological background with MG [3].

MG has a clear autoimmune substrate [1] with a primary pathogenic role for the thymus. Both experimental autoimmune MG animal model and analysis of circulating antibodies in MG patients indicate a T-cells dependent mechanism [1,12] originating from the thymoma [3]. Transgenic MRL/lpr mice, used as an experimental model for autoimmune diseases, have immunopathologic features partially similar to those described in MG. Interestingly, they were found with increased T-cell infiltration and selectively decreased α-gustducin positive taste receptor cells in taste buds. Alpha-gustducin, a G-protein subunit co-expressed with almost all T1 and T2 receptors in GPCR, is responsible for sweet, bitter and umami perception [8]. As expected, MRL/lpr transgenic mice displayed diminished gustatory nerve response to bitter and sweet compounds, and reduced behavioral response to bitter, sweet and umami substances, while their salt and sour taste cells characteristics and behavior did not differ from control animals [12]. All together, these observations suggest the involvement of α-gustducin in MG-related (sweet) dysgeusia. Furthermore, various antibodies associated with thymic abnormalities in MG are reported without any clearly defined contribution to the pathology and the severity of the disease [1,13]. We hypothesize that one of them could target α-gustducin positive receptor cells, disturb taste signaling and that way elicit dysgeusia in MG [2].

Our case and recent reports indicate thymoma-associated MG with AChRAb as a plausible etiology of sweet taste loss, possibly due of an autoimmune mechanism targeting GPCRs taste cells expressing α-gustducin as the underlying mechanism. We strongly emphasize that unexplained selective sweet taste alteration, associated with possibly overlooked mild MG, as in our patient, deserves thorough workup with a high degree of suspicion for a paraneoplastic origin.

## Abbreviations

AChRAb: Anti-acetylcholine receptor antibody; CSF: Cerebrospinal fluid; DSS: Dysgeusia subjective scale; GPCRs: G-protein-coupled receptor cells; ICU: Intensive care unit; MG: Myasthenia gravis; MGFA: Myasthenia gravis Foundation of America; MRI: Magnetic resonance imaging.

## Competing interests

The authors declare that they have no competing interests.

## Authors’ contributions

Study concept and design: C, T, L and K. Acquisition of data: C, T, Z, L, and K. Analysis and interpretation of data: C, L and K. Drafting of the manuscript: C and K. Critical revision of the manuscript for important intellectual content: C, Z, L and K. Administrative, technical, and material support: C and K. Study supervision: C and K. All authors read and approved the final manuscript.
